# Heart Failure and Multimorbidity in Asia

**DOI:** 10.1007/s11897-023-00585-2

**Published:** 2023-02-22

**Authors:** Nathalie Ang, Chanchal Chandramouli, Kelvin Yiu, Claire Lawson, Jasper Tromp

**Affiliations:** 1 Saw Swee Hock School of Public Health, The National University of Singapore (NUS), 12 Science Drive 2, Singapore, #10-01117549 Singapore; 2 Duke-NUS Medical School, Singapore, Singapore; 3 National Heart Center, Singapore, Singapore; 4 Division of Cardiology, Department of Medicine, The University of Hong Kong, Hong Kong, China; 5 Division of Cardiology, Department of Medicine, The University of Hong Kong-Shenzhen Hospital, Shenzhen, China; 6 University of Leicester, Leicester, UK; 7 Department of Cardiology, University Medical Centre Groningen, University of Groningen, Groningen, Netherlands

**Keywords:** Heart failure, Multimorbidity, Asia

## Abstract

**Purpose of the Review:**

Multimorbidity, the presence of two or more comorbidities, is common in patients with heart failure (HF) and worsens clinical outcomes. In Asia, multimorbidity has become the norm rather than the exception. Therefore, we evaluated the burden and unique patterns of comorbidities in Asian patients with HF.

**Recent Findings:**

Asian patients with HF are almost a decade younger than Western Europe and North American patients. However, over two in three patients have multimorbidity. Comorbidities usually cluster due to the close and complex links between chronic medical conditions. Elucidating these links may guide public health policies to address risk factors. In Asia, barriers in treating comorbidities at the patient, healthcare system and national level hamper preventative efforts.

**Summary:**

Asian patients with HF are younger yet have a higher burden of comorbidities than Western patients. A better understanding of the unique co-occurrence of medical conditions in Asia can improve the prevention and treatment of HF.

## Background

Heart failure (HF) affected approximately 64.3 million individuals globally in 2017 [1]. The American Heart Association (AHA) estimated annual spending of USD351.2 billion from 2014 to 2015 on HF in the USA alone [2]. Therefore, HF greatly strains the health system [3, 4]. Unfortunately, HF’s prognosis remains grim [5, 6]: the 5-year mortality risk after the diagnosis is 50%, which is worse than many types of cancer [5, 7, 8]. Despite Asia being home to most of the world’s population, most HF data is from Western Europe and North America [9].

Due to widespread population ageing and an increasing comorbidity burden, more people live with chronic diseases [10–12]. Individuals with comorbidities often have multimorbidity, defined as having two or more chronic conditions. Comorbidities often share intimate links due to shared disease pathways or pathophysiological processes. People with one comorbidity are often at a higher risk of developing the next. For example, people with diabetes are at an increased risk of developing chronic kidney disease (CKD) [13–16]. Importantly, people with more comorbidities are more likely to develop HF [17].

In Asia, multimorbidity is becoming increasingly common [18]. In some Indian states, multimorbidity affects almost 10% of individuals [19, 20]. In China, 52% of middle-aged individuals have multimorbidity [21]. Data on patients with *prevalent* HF showed that approximately two in three Asian patients have at least two comorbidities apart from HF. Chiefly, multimorbidity patterns in patients with HF are associated with worse outcomes [22].

The prevalence of multimorbidity varies by region, highlighting the influence of genetics, socioeconomic determinants and cultural and environmental factors [10, 11, 22, 23]. Many Asian countries, such as Thailand, Singapore and China, underwent a rapid economic development in the past few decades. The rapid economic development led to a ‘Westernization’ of diets and an increase in sedentary lifestyle. These rapid changes occurred in a population where many, especially older, people did not attain formal education. This combination has caused region-specific challenges in patients with a high disease burden but low (health) literacy. Disentangling the shared links between comorbidities can help identify a single or a limited number of common risk factors. These can serve as early primary and secondary prevention targets, reducing the number of quality-adjusted life-years lost, potentially decreasing costs and the burden on health systems. Unfortunately, there are significant barriers to diagnosing and treating risk factors in Asia, especially in lower- and middle-income countries. Therefore, this review will discuss (1) regional patterns of comorbidities and multimorbidity in Asian patients with HF compared with patients from the west and (2) barriers to implementation of treatment and prevention.

## Heart Failure Registries in Asia

**Fig. 1:**
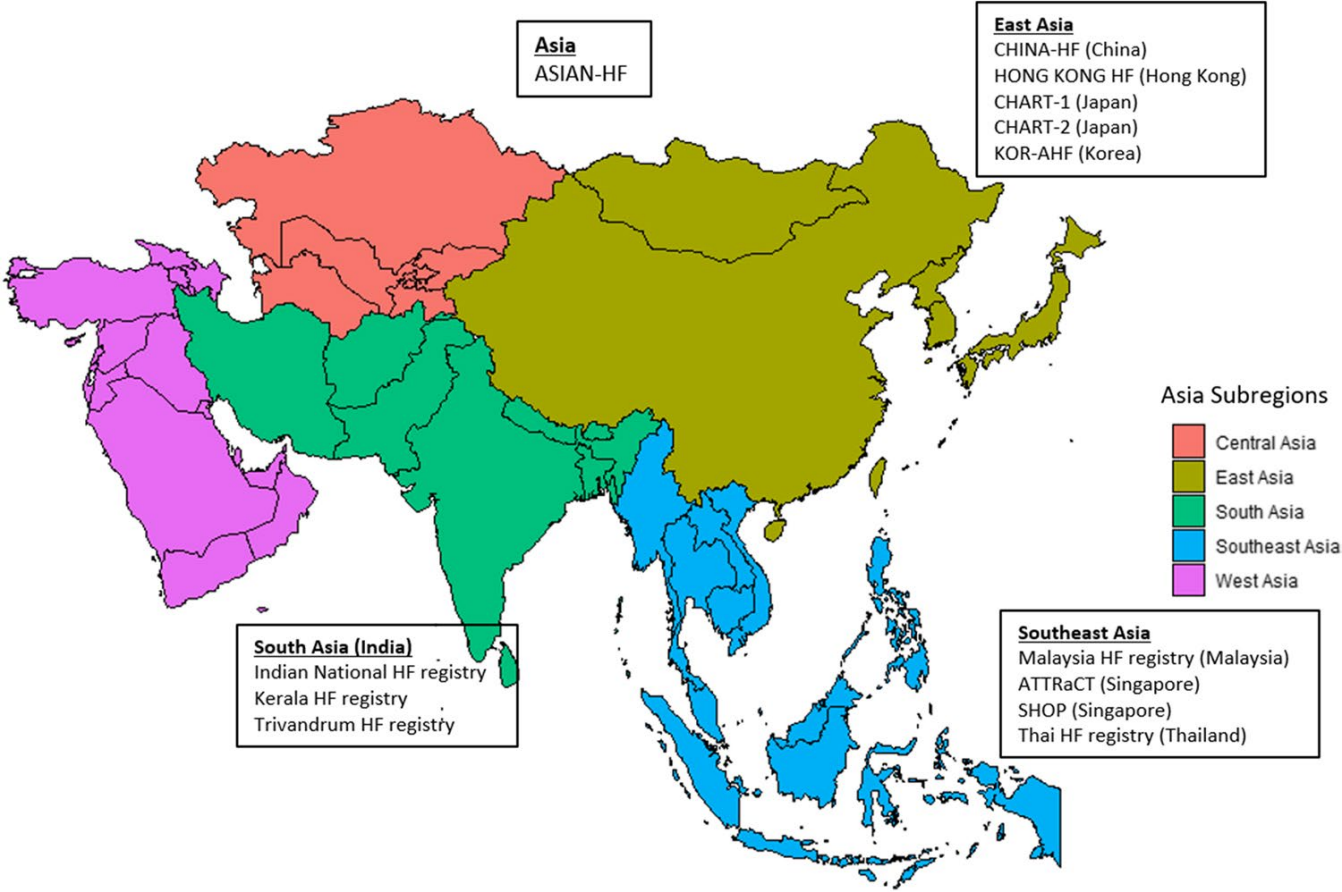
A map of Asia showing Asian countries coloured according to geographical regions with a non-exhaustive list of registries

In contrast to the extensive data regarding HF in Western nations, epidemiologic data are still scarce in Asian patients with HF. Figure 1 shows a non-exhaustive list with previous and current HF registries in Asia. This overview highlights the increasing efforts in collecting data on Asian patients with HF but also emphasizes the significant gap. Some of the first HF registries in Asia were the CHART studies [24] in Japan and the KOR-AHF registry [25] in South Korea. The Asian Sudden Cardiac Death in Heart Failure registry (ASIAN-HF) was the first multinational regional registry prospectively to include Asian patients with both HF with reduced ejection fraction (HFrEF) and HF with preserved ejection fraction (HFpEF) patients. This registry included data from 11 Asian regions (China, Hong Kong, India, Indonesia, Japan, Korea, Malaysia, the Philippines, Singapore, Taiwan and Thailand) [26]. There have been various country-specific studies, such as the Kerala-HF registry [27] in India, the ATTRaCT and SHOP studies in Singapore [28, 29] and CHINA-HF [30] in China. However, there are still few large HF registries, including many Asia regions (Central Asia and West Asia) or countries (Indonesia, Malaysia and Thailand), which are crucial to determining the HF trend and risk factors [31–34].

## Prevalence of Leading Comorbidities in Patients with Heart Failure from Asia Compared to Western Europe and North America

Because the comorbidity burden differs significantly between HFrEF and HFpEF [35, 36], the comorbidity patterns of patients with HFrEF and HFpEF are discussed separately.

### Heart Failure with a Reduced Ejection Fraction (HFrEF)

The Prospective Comparison of ARNI with ACEI to Determine Impact on Global Mortality and Morbidity in Heart Failure (PARADIGM-HF) trial was among the first randomized controlled clinical trials to include a significant number of patients with HFrEF from the Asia–Pacific region, which included China, Hong Kong, India, Israel, South Korea, Malaysia, the Philippines, Singapore, Thailand and Taiwan. Table 1 shows that patients in PARADIGM-HF from the Asia–Pacific region were younger, had a lower body mass index (BMI), lower prevalence of atrial fibrillation (AF) and a lower prevalence of hypertension than patients from Western Europe or North America.

**Table 1:** Summary table of regional differences among patients with heart failure with reduced ejection fraction (HFrEF) in trials and registries

	*Trial*			*Registries*								
	*PARADIGM-HF* [37]			*ASIAN-HF* [38]			*CHART-2* [39]	*KOR-AHF* [40]	*CHINA-HF* [30]	*Trivandrum HF registry* [41]	*ESC-HF-LT* [42]	*CHAMP-HF* [43]
	AP	NA	WE	Northeast Asia	South Asia	Southeast Asia	Japan	South Korea	China (POC)	India	Europe	NA
*N*	1487	1680	602	1658	1436	2182	730	3105	211	752	7173	3497
Mean age (years)	58	65	68	63	58	59	67	66	62	62	65	66
Mean BMI (kg/m^2^)	24	31	29	24	25	26	23	23	23	-	28	30
Women (%)	20	17	18	25	24	18	23	39	32	27	29	29
AF (%)	17	40	44	30	4	18	38	29	-	13	38	36
CAD/MI (%)	58	62	58	38	51	59	39	19	17	-	-	-
Hypertension (%)	48	84	63	48	38	64	85	59	43	59	58	82
CKD (%)	27	52	45	39	37	53	-	-	8	17	18	26
Diabetes (%)	35	49	36	32	37	49	38	42	20	55	32	41
COPD (%)	6	23	16	11	5	9	-	11	5	17	14	35
Cancer (%)	1	14	10	7	0	2	12	-	-	-	-	11

*AF*, atrial fibrillation; *AP*, Asia–Pacific; *ASIAN-HP*, the Asian Sudden Cardiac Death in Heart Failure registry; *BMI*, body mass index; *CAD*, coronary artery disease; *CHAMP-HF*, the Change the Management of Patients with Heart Failure registry; *CKD*, chronic kidney disease; *COPD*, chronic obstructive pulmonary disease; *ESC-HF-LT*, the European Society of Cardiology Long-Term registry; *NA*, North America; *PARADIGM-HF*, the global Prospective Comparison of ARNI with ACE inhibitor to Determine Impact on Global Mortality and Morbidity in Heart Failure Trial; *WE*, Western Europe

The ASIAN-HF was one of Asia’s first multinational ‘real-world’ HF registries highlighting the unique characteristics of Asian patients with HFrEF. This registry prospectively enrolled more than 6300 patients from 46 centres across 11 Asian regions [26, 38, 44]. The registries’ results highlighted the young age of Asian patients with HF. Table 1 shows that the mean age of patients from South Asia was only 58 years compared to 65 or 66 in the European Society of Cardiology Long-Term registry (ESC-HF-LT) and Change the Management of Patients with Heart Failure registry (CHAMP-HF) registry, respectively. Importantly, the ASIAN-HF registry highlighted the considerable regional heterogeneity within Asia: differences in age and comorbidities were as significant among Asian regions as between Asian and Western European registries (Table 1).

Despite their relative youth, Asian patients had a high comorbidity burden [38]. Notably, Southeast Asian patients were younger but had the highest prevalence of comorbidities, such as CKD and diabetes, compared to patients from Northeast Asia and South Asia (India) [22]. Data from other Asian registries mirrored these data. Japanese and Korean patients in the CHART-2 and KOR-AHF [39, 40] registries, respectively, had a similar mean age as patients from European [42] or North America [45]. They were older than South or Southeast Asian patients. Notably, patients from Korea and Japan had a high prevalence of hypertension, which is also true for their general population [46]. In contrast, patients with HFrEF from CHINA-HF [30] and the Trivandrum HF registry [41] (India) were younger than patients in Northeast Asian registries. Notably, Indian patients had a very high prevalence of diabetes [41], highlighting the significant burden of this comorbidity on that region.

### Heart Failure with a Preserved Ejection Fraction (HFpEF)

PARAGON-HF was the first multinational phase 3 HFpEF trial, including a significant number of patients from Asia. Regional differences in PARAGON-HF largely mirrored those in PARADIGM-HF: patients from Asia were younger yet had a similar high comorbidity burden as those from Western Europe and North America [47]. Notably in PARAGON-HF was the high prevalence of diabetes in Asia despite having a lower mean body mass index (BMI) than other regions.

Table 2 shows that differences among regional registries mirror differences in PARAGON-HF. The age gap between Asian and Western countries was larger than in HFrEF. Patients from South Asia and Southeast Asia in ASIAN-HF were almost a decade younger than patients from the Swedish Heart Failure registry (SWEDE-HF) or the Framingham Heart Study (FHS). Despite their relative youth, these patients had a high comorbidity burden. A previous study investigating younger patients with HFpEF in Asia highlighted that younger Asian patients were more likely to be men, of Malay or Indian origin, and with a high prevalence of obesity [48]. The worse outcomes of younger patients than age- and sex-matched controls confirmed that these patients truly had HF [48]. The unique characteristics of younger patients with HFpEF (male, Asian, obese) were further confirmed in a combined study of the Treatment of Preserved Cardiac Function Heart Failure with an Aldosterone Antagonist trial (TOPCAT), Candesartan in Heart failure: Assessment of Reduction in Mortality and morbidity study (CHARM-Preserved) and Irbesartan in Heart Failure with Preserved Ejection Fraction Study (I-PRESERVE) studies [49].

**Table 2:** Summary table of regional differences among patients with heart failure with preserved ejection fraction (HFpEF) in trials and registries

	*Trial*			*Registries*								
	*PARAGON-HF* [47]			*ASIAN-HF* [38]			*CHART-2* [39]	*KOR-HF* [40]	*CHINA-HF* [30]	*Trivandrum HF registry* [41]	*SWEDE-HF* [50]	*FHS* [51]
	AP	NA	WE	Northeast Asia	South Asia	Southeast Asia	Japan	South Korea	China (POC)	India	WE	NA
*N*	762	559	1390	543	252	409	2154	1295	343	190	9140	220
Mean age (years)	72	74	75	72	63	67	72	72	71	59	77	80*
Mean BMI (kg/m^2^)	28	32	30	26	28	28	23	24	24	-	28	27
Women (%)	50	47	52	51	47	50	61	62	52	44	54	65
AF (%)	34	29	36	36	7	31	51.8	41	-	24	55	29
MI (%)	23	24	21	-	-	-	26.9	9	16	-	-	-
Hypertension (%)	92	97	94	75	40	83	91.2	67	60	55	56	59
CKD (%)	54	37	46	44	41	60	-	-	6	17	55	-
Diabetes (%)	44	49	39	39	29	62	33.8	34	20	48	25	22
COPD (%)	-	-	-	10	5	11	-	13	11	17	-	-
Cancer (%)	-	-	-	8	1	2	15.8	-	-	-	-	-

*AF*, atrial fibrillation; *AP*, Asia–Pacific; *ASIAN-HP*, the Asian Sudden Cardiac Death in Heart Failure registry; *BMI*, body mass index; *CAD*, coronary artery disease; *CKD*, chronic kidney disease; *COPD*, chronic obstructive pulmonary disease; *FHS*, Framingham Heart Study; *NA*, North America; *MI*, myocardial infarction; *PARAGON-HF*, Prospective Comparison of Angiotensin Receptor Neprilysin Inhibitor with angiotensin Receptor Blocker Global Outcomes; *SWEDE-HF*, Swedish Heart Failure Registry; *WE*, Western Europe

^*^Age presented as median

## Regional Differences in Patterns of Multimorbidity

Several studies have highlighted the burden, patterns and consequences of HF patients with multimorbidity [20, 52]. In the ASIAN-HF registry, the latent class analysis revealed five multimorbidity groups with distinct geographic distributions across Asia, each affecting patients’ quality of life and health outcomes differently. First, there was an *elderly/AF* (old and more atrial fibrillation (AF)) pattern, primarily consisting of patients from Hong Kong, Japan and Korea. Second is a metabolic (obese, hypertensive and diabetic) pattern, most prevalent in Malaysia, the Philippines, Singapore and Taiwan. Third is a *young* (younger and lesser comorbidities) pattern, most prevalent in China, India, Japan, Korea and Thailand. Fourth is an *ischemic* (coronary artery disease (CAD) and ischemic aetiology) pattern, most prevalent in India, Indonesia and Malaysia. Fifth is a *lean-diabetic* (diabetic and low BMI) pattern, most commonly found in Hong Kong, Malaysia and Singapore. The lean-diabetic (predominantly type II diabetes) seemed unique to Asia and was not seen in previous studies investigating multimorbidity. For example, Gulea et al. identified five multimorbidity patterns in the USA: *low-burden* (minimal comorbidities), *metabolic-vascular* (diabetic, obese and vascular disease), *anaemic* (cancer and depression), *ischemic* (oldest) and *metabolic* (young, diabetic and obese) [53]. In Spain, a previous study identified six groups which largely mirror those found in the USA but did not include a lean-diabetic phenotype [54].

The ‘thrifty gene’ hypothesis might explain the existence of a lean-diabetic phenotype. Southeast Asia has undergone a rapid epidemiological transition due to economic development and westernization of diet [55]. The ‘thrifty gene’ hypothesis suggests that individuals who can easily store extra energy had an evolutionary advantage during previous famines [56]. The existence of a lean-diabetic phenotype might further be explained by the greater propensity of Asians to store fat in the visceral space. In a study investigating the nexus between BMI and abdominal fat, patients with a low BMI but high waist-to-height ratio had the worst quality of life and the highest prevalence of diabetes, emphasizing the unique role of excess visceral fat in Asian patients with HF [57]. A study investigating the role of epicardial fat, which is part of the visceral fat depot, found that increased levels of epicardial fat were associated with adverse cardiac remodelling and fibrosis [58]. This might also explain why the prevalence of diabetes is significantly higher in Asians than in White patients for any given BMI [59].

Recognizing the shared links between comorbidities and identifying common risk factors can guide public health investments and preventative efforts. A previous study, performed in the Australian Longitudinal Study on Women’s Health (ALSWH), characterized the longitudinal progression of three conditions and multimorbidity. The authors found that the odds of developing new conditions were significantly higher in women who already had pre-existing comorbidities than those without any [60]. These critical results highlighted that comorbidities cluster over time and that progression of multimorbidity is likely not linear but exponential. A longitudinal study using data from general practices in the UK showed similar results [61]. In this study, diabetes was the most common initial onset condition, and South Asians were at a significantly higher risk of developing diabetes [61]. Collectively, these results highlight the importance of identifying shared links between comorbidities and emphasize the increased risk of (South and Southeast) Asians developing multimorbidity.

## Treatment of Comorbidities in Asia: Barriers to Implementation

**Fig. 2:**
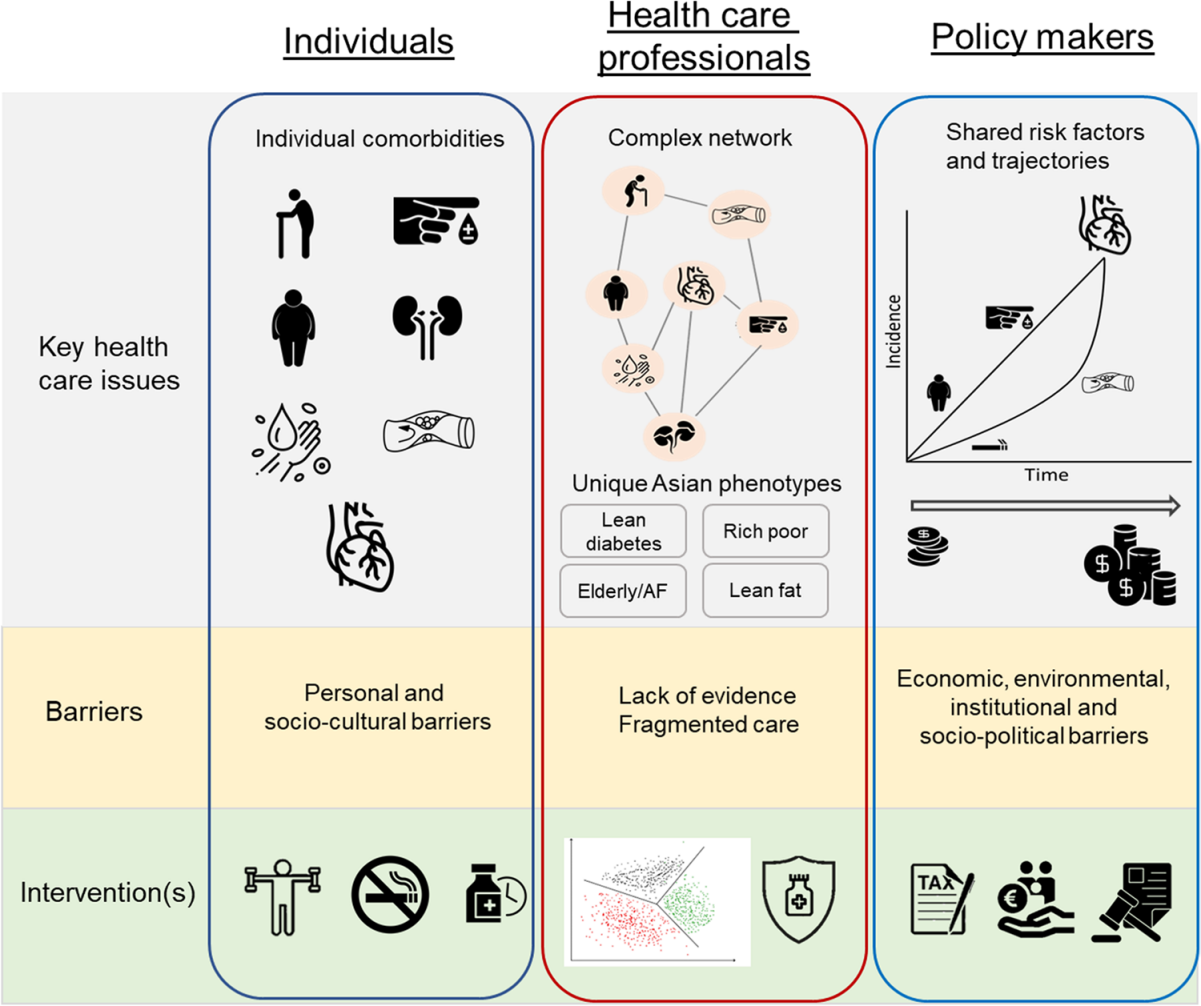
Summary figure of various healthcare issues, barriers and possible solutions at different levels of the health system. Key barriers in Asian population include socio-cultural beliefs and often low education/health literacy of (elderly) patients, fragmented and weak primary healthcare systems and high out-of-pocket costs for patients and carers

The first step is to recognize the importance of multimorbidity and different early onset conditions (Fig. 2). Adequate treatment and prevention of comorbidities to reduce the prevalence of multimorbidity and HF are the logical consequence, but there are significant patient, healthcare professional and policy-level barriers in Asia that may inhibit adequate care and prevention.

In many Asian countries, patients often have insufficient knowledge and understanding of chronic disease due to low educational levels, persistent myths and beliefs and financial challenges. As a result, (often older) patients tend to believe in natural remedies and traditional herbal practitioners as cheaper alternatives to (‘Western’) medicine, which often leads to misconceptions and worse health outcomes [62, 63]. In addition, Asian patients seem to face even more significant difficulties in adopting to a healthier lifestyle than elsewhere. While attempting to modify their habits, most reverted to their original lifestyles, and one of the most difficult habits to change was their dietary regimen [63]. Improved patient education on the importance of a healthy lifestyle, smoking cessation and medication adherence are vital interventions for these issues. Policy makers should promote low-cost healthy diets (lower salt, sugar and oil content in foods) and increased physical activities to alleviate the CVD epidemic. These interventions are often highly cost-efficient [64, 65].

Implementing preventive policies and chronic disease management in a financially sustainable way requires strengthening primary care systems to provide low-cost personalized care by providers close to local communities [66]. In South and Southeast Asia, many countries have a weak primary care system. Primary care physicians often operate as single private practices and have a limited role in chronic disease management and prevention [67]. Instead, patients often self-refer to specialized hospitals, leading to fragmented care of multimorbidity, increased costs and additional financial barriers to care.

Strengthening primary care would require a policy-level commitment with sufficient financial investments to enable primary care physicians to manage chronic diseases. This can further be supported by digital technologies, which enable the integration of electronic health records with secondary and tertiary care providers, which is key for chronic disease management.

## Conclusion

Asian patients with HF are up to a decade younger than Western patients. Despite their relative youth, multimorbidity is common. Having two or more comorbidities is associated with an increased risk of developing HF and worsens outcomes and quality of life of people living with HF. Asia is home to a unique lean-diabetic multimorbidity HF phenotype with worse outcomes. A better understanding of the unique co-occurrence of medical conditions in Asia can improve the prevention and treatment of HF. However, there remains a significant unmet need to address barriers in treating comorbidities at the patient, healthcare system and national level.
